# High Diversity and Ancient Common Ancestry of Lymphocytic Choriomeningitis Virus

**DOI:** 10.3201/eid1607.091902

**Published:** 2010-07

**Authors:** Cesar G. Albariño, Gustavo Palacios, Marina L. Khristova, Bobbie R. Erickson, Serena A. Carroll, James A. Comer, Jeffrey Hui, Thomas Briese, Kirsten St. George, Thomas G. Ksiazek, W. Ian Lipkin, Stuart T. Nichol

**Affiliations:** Author affiliations: Centers for Disease Control and Prevention, Atlanta, Georgia, USA (C.G. Albariño, M.L. Khristova, B.R. Erickson, S.A. Carroll, J.A. Comer, T.G. Ksiazek, S.T. Nichol);; Columbia University, New York, New York, USA (G. Palacios, J. Hui, T. Briese, W.I. Lipkin);; New York State Department of Health, Albany, New York, USA (K. St. George)

**Keywords:** Genetic diversity, viruses, lymphocytic choriomeningitis virus, phylogeny, LCMV, rodent-borne viruses, research

## Abstract

*Lymphocytic choriomeningitis virus* (LCMV) is the prototype of the family *Arenaviridae*. LCMV can be associated with severe disease in humans, and its global distribution reflects the broad dispersion of the primary rodent reservoir, the house mouse (*Mus musculus*). Recent interest in the natural history of the virus has been stimulated by increasing recognition of LCMV infections during pregnancy, and in clusters of LCMV-associated fatal illness among tissue transplant recipients. Despite its public health importance, little is known regarding the genetic diversity or distribution of virus variants. Genomic analysis of 29 LCMV strains collected from a variety of geographic and temporal sources showed these viruses to be highly diverse. Several distinct lineages exist, but there is little correlation with time or place of isolation. Bayesian analysis estimates the most recent common ancestor to be 1,000–5,000 years old, and this long history is consistent with complex phylogeographic relationships of the extant virus isolates.

## Abstract 2

The diversity of these viruses has practical implications for the design of molecular diagnostic assays.

The rodent-borne arenaviruses (family *Arenaviridae*) are enveloped viruses with bisegmented RNA genomes that include several causative agents of hemorrhagic fevers in the New World and Africa (*1*). The large (L) genome RNA segment encodes the virus polymerase L and the Z protein, whereas the small (S) genome RNA segment encodes the nucleocapsid protein (NP) and glycoprotein precursor (GPC). The prototypic arenavirus, *Lymphocytic choriomeningitis virus* (LCMV), is distributed worldwide (due to its association with rodents of the species *Mus musculus*). This virus is typically associated with mild, self-limited, or asymptomatic infections in immunocompetent persons, but infections can lead to aseptic meningitis (*1*). In immunocompromised persons, LCMV exposure may result in serious systemic infections and death (*2*). Prenatal infection can cause spontaneous abortion or severe birth defects, including hydrocephalus, chorioretinitis, blindness, or psychomotor retardation (*3**,**4*).

Recent clusters of fatal disease in organ transplant recipients have focused new attention on the potential for iatrogenic transmission of LCMV. In December 2003 and April 2005, recipients of solid-organ transplants linked to single donors, died of unexplained infections. LCMV was implicated after the results of viral culture and electron microscopy triggered specific immunohistochemical and molecular tests for arenaviruses (*2*). In the 2005 cluster, a pet hamster that had been introduced into the donor’s household was infected with the same virus that was later detected in the recipients (*5*). In early 2007, three patients who received visceral transplants on the same day from a single donor died of a febrile illness 4–6 weeks after transplantation. Unbiased high-throughput sequencing yielded sequences that identified a novel LCMV-related arenavirus (*6*). However, phylogenetic characterization was limited by the paucity of available sequences deposited in public databases. In April 2008, a public health investigation showed evidence of acute LCMV infection in 2 transplant recipients who had received kidneys from a common donor. Both patients died 4 and 10 weeks after transplantation despite intensive supportive care (*7*).

In spite of the increasing awareness of the public health importance of LCMV, little is known about the genetic diversity or relationships of LCMVs found in various parts of the world. Previous studies have suggested that nucleotide sequence divergence is high, up to 22% between some LCMVs (*8**–**10*). In the current study, we investigate the genetic diversity of 29 LCMVs, and infer from those sequences a history reaching back >1,000 years, findings consistent with the existing complex virus phylogeographic patterns.

## Materials and Methods

Most of the sequences included in the alignments correspond to complete segment sequences. However, some short sequences, such as those from Kodoko virus, were also included in the analysis. This approach was taken to obtain the best reconstruction of the evolutionary history of the taxa (viruses, in our case) by using the maximum number of informative sites available (*11**–**13*). Although the validity of including missing data has been debated in the past, more recent studies have shown that even highly incomplete taxa can be placed accurately within the phylogeny (*11**,**12**,**14*).

The appropriateness of this approach was further examined by running several preliminary analyses. Initially, only full-length segment sequences were analyzed. Once the relationships between taxa and rate estimates were established, partial sequences (e.g., Kodoko virus) were also added to the analyses. No rate shifts were observed nor were any strongly supported phylogenetic relationships obscured. As a result, the tree figures shown in this report were based on the dataset including both whole segment and partial segment sequences.

From virus collections at the Centers for Disease Control and Prevention, the New York State Department of Health, Columbia University, and the World Reference Center of Emerging Viruses and Arboviruses (University of Texas Medical Branch), we selected 12 LCMVs for genetic characterization; origins spanned >70 years with broad geographic distribution (Table 1). Included in the study were representative virus stocks of the classic WE LCMV strain. This strain was originally isolated from a meningoencephalitis patient in New York in 1935 (*15*). In that era, virus isolation and passage were performed by intracranial inoculation into mice, which resulted in isolates that had multiple passages in mice as part of their passage history. Although the WE strain is used extensively in immunobiology experiments, the exact passage history of these viruses has been poorly documented. We located 2 old stocks of WE, 1 lyophilized in 1950 with a record of 7 passages in mouse brain, and 1 lyophilized in 1960 with a record of 7 passages in mouse brain and virus plaque purification (Table 1). In the 1940s, the WE LCMV strain was transferred to the University of British Columbia from the Rockefeller Institute in New York, only to be returned to the New York State Department of Health some years later. This substrain of WE LCMV became known as UBC (*16*). The 2 lyophilized vials were both labeled as UBC WE LCMV. Two other early LCMV isolates were also found. The Douglas-4707 and WHI-5107 strains were isolated by intracranial inoculation of suckling mice from the cerebrospinal fluid of patients in New York who had aseptic meningitis in 1947 and 1949, respectively. These viruses were recovered from lyophilized stocks prepared in 1960 and 1950, respectively (*17*), and represent some of the oldest low passage LCMV stocks still in existence.

**Table 1:** Origins of lymphocytic choriomeningitis virus strains analyzed*

Strain (other names)	Collection date (passage history)	Associated case (sample source)	GenBank accession no.
Massachusetts-2008 (811316, 200802972)	Massachusetts, USA, 2008	Transplant case (human blood)	FJ607022,† FJ607031†
Dandenong	Former Yugoslavia, 2006	Transplant case (human liver)	EU136039, EU136038
Rhode Island-2005 (810850, 200501927)	Rhode Island, USA, 2005	Transplant case (hamster kidney)	FJ607021,† FJ607030†
Ohio-2005(810896, 200504261)	Ohio, USA, 2005	Transplant case (hamster kidney)	FJ607026,† FJ607037†
Michigan-2005 (810885, 200504219)	Michigan, USA, 2005	House rodent infestation (mouse spleen)	FJ607023,† FJ607032†
California-2003 (810366, 200312154)	California, USA, 2003	Congenital infection (human CSF)	FJ607019,† FJ607028†
Wisconsin-2003 (810362, 200312181)	Wisconsin, USA, 2003	Transplant case (human CSF)	FJ607027,† FJ607038†
Lyles (810935, Georgia-1984)	Georgia, USA, 1984	House rodent infestation (human CSF)	FJ607020,† FJ607029†
Douglas-4707 (810938, NY-H938)	New York, USA, 1947 (stock lyophilized in 1960; 1 passage in VE6 in 2005)	Human CSF	FJ607024,† FJ607035†
WHI-5107 (810906, NY-H906)	New York, USA,1949 (stock lyophilized in 1950)	Human CSF	FJ607033†
WE-UBC-57135 (810940, NY-H940)	New York, USA,1935 (7 passages in MB, plaque purified; stock lyophilized in 1960; 1 passage in VE6 in 2005)	Human specimen	FJ607025,† FJ607036†
WE-UBC-A337 (810909, NY-H909)	New York, USA, 1935 (7 passages in MB, stock lyophilized in 1950)	Human specimen	FJ607034†
Bulgaria	Bulgaria, 1956? (WRCEVA collection at UTMB)	?	GQ862981,† GQ862982†
M1, M2	Austria, 2005	Infection of mouse colony (mouse spleen)	AB261990, AB261991
LE	France, 2006	Congenital infection (amniotic fluid)	EF164923
Marseille	France, 2004	House rodent infestation (mouse kidney)	DQ286932, DQ286931
CH5692, CH5871	Germany, 1999; Germany, 2000	Infection of monkey colony (monkey spleen and serum)	AY363903, AF325214, AF325215,
CHV1, CHV2, CHV3	Oklahoma, USA,1986	Infection of monkey colony (monkey liver)	U10157, U10158, U10159
MX	Slovakia, 1998	Persistently infected cell line	EU195888, EU195889
Yale (Y)	Connecticut, USA, 1977	Mouse	DQ118959
WE	New York, USA, 1935	First recognized aseptic meningitis by LCMV	M22138, AF004519
Armstrong	Missouri, USA, 1933	St. Louis encephalitis epidemics	AY847351, M20869
CABN, GR01, SN05	Spain, 2004	Wild mice (mouse lungs)	FJ895882, FJ895883, FJ895884

*CSF, cerebrospinal fluid; MB, mouse brains; WRCEVA, World Reference Center of Emerging Viruses and Arboviruses; UTMB, University of Texas Medical Branch; LCMV, lymphocytic choriomeningitis virus. ? represents the uncertainty of this virus origin. †New sequences.

The Lyles LCMV strain was isolated in Vero cells from the cerebrospinal fluid (CSF) of a 58-year-old woman from Winder, Georgia, who had nonfatal aseptic meningitis and a history of exposure to mice in her home (*18*). Similarly, the Michigan 2005 LCMV strain was isolated in 2005 from a mouse captured around the home of a 46-year-old woman with a diagnosis of acute meningitis and mild pancreatitis (*19*). The California 2003 LCMV was isolated in 2003 from the CSF of a congenitally infected infant with severe neurologic sequelae, including hydrocephalus, chorioretinitis, blindness, and developmental delay (*20*). The other LCMV isolates were from investigations of clusters of deaths and severe illness in transplant recipients associated with LCMV infection from transplanted organs. Four isolates were obtained during 2003–2008 from infected transplant recipients or rodents suspected of being involved in the exposure of the transplant donor in various locations in the United States (*2**,**5**,**7*). The Dandenong isolate was obtained from the liver of a patient who died after transplantation in Australia; the donor was suspected to have acquired the infection while traveling in the Balkans shortly before death and the harvesting of his organs (*6*). Finally, the isolate from Bulgaria (1956) is strongly suspected of being the first isolate obtained in Bulgaria from a case-patient with confirmed lymphocytic choriomeningitis (*21*).

RNA was extracted either directly from virus stocks or from supernatant harvested from infected cell cultures. A 300-µL aliquot of virus stock or cell culture supernatant was mixed with 900 µL of TRI Reagent (Molecular Research Center, Inc., Cincinnati, OH, USA) and 240 µL of chloroform and extracted according to standard protocols. The nucleic acid obtained was reverse transcribed and amplified by PCR; a total of 12 LCMV S segment sequences and 10 LCMV L segment sequences were amplified and sequenced by dideoxy-sequencing (Applied Biosystems, Foster City, CA, USA). We were unable to amplify by PCR the L segments of LCMV strains WHI-5107 and UBCA337 from the original virus ampoules, and the viruses were found to be no longer viable. The origins of 16 LCMV isolates for which sequences were already available, and that were included in the study, are also shown in Table 1. Multiple sequence alignments were generated using Multiple Alignment with Fast Fourier Transform (*22*) in SeaView (*23*) and sequence diversity was calculated by using molecular evolutionary genetics analysis (MEGA) 4 (*24*). Bayesian phylogenetic analyses of the sequence differences among the S and L segments of LCMV and Kodoko viruses were conducted using the BEAST, BEAUti and Tracer analysis software packages (*25*). Preliminary analyses were run for 10,000,000 generations with the Hasegawa, Kishino, and Yano + Γ nucleotide substitution model to select the clock and demographic models most appropriate for the S and L data sets. An analysis of the marginal likelihoods indicated that the relaxed lognormal molecular clock and constant population size model was decisively chosen (log_10_ Bayes factors = 3.032 for S segment; 13.472 for L segment). Final data analyses included Markov chain Monte Carlo chain lengths of 20,000,000–480,000,000 generations, with sampling every 1,000 states.

## Results

**Figure 1:**
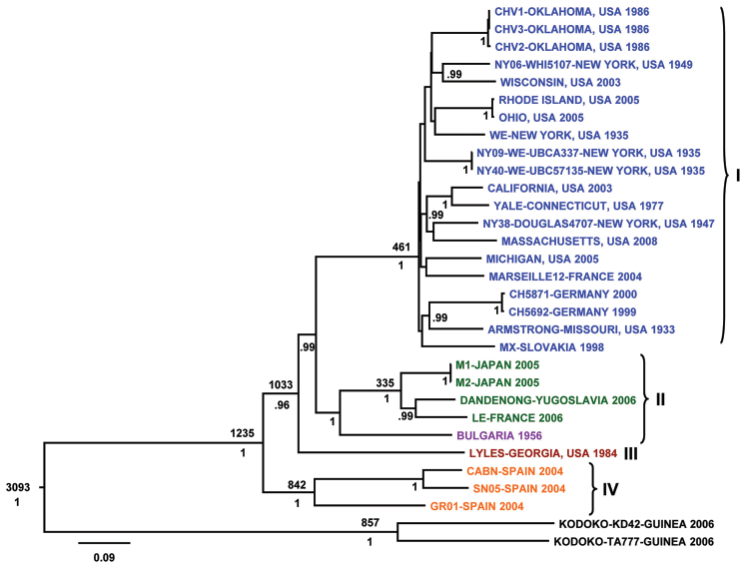
Bayesian coalescent analysis of lymphocytic choriomeningitis virus (LCMV) based on the small (S) gene segment. The maximum clade credibility tree generated from analysis of available LCMV S segment sequences is shown. Branch lengths are proportional to the number of substitutions/site/year. Depicted at the main nodes are the time to most recent common ancestor estimates (TMRCA) based on Bayesian coalescent analysis of the virus sequences and isolation dates without inclusion of the Bulgarian strain for which no reliable isolation date was available. Posterior probabilities are listed below the branches for supported nodes. Scale bar indicates nucleotide substitutions per site.

**Figure 2:**
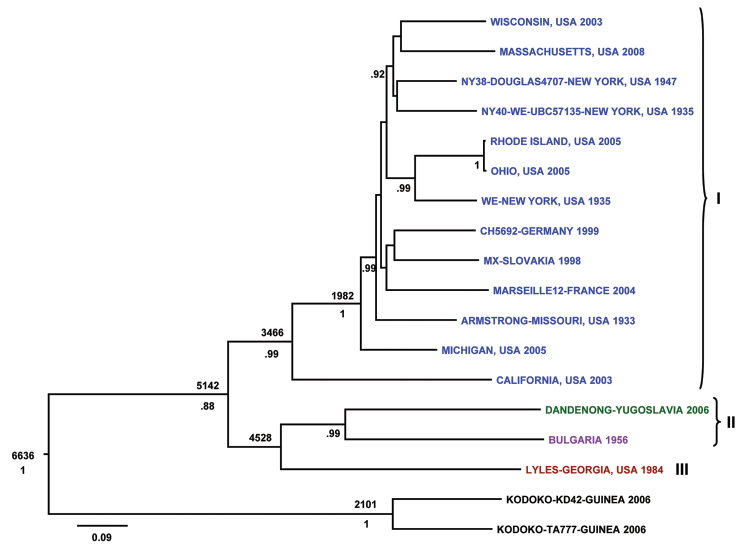
Bayesian coalescent analysis of lymphocytic choriomeningitis virus (LCMV) based on the large (L) gene segment. The maximum clade credibility tree generated from analysis of available LCMV L segment sequences is shown. Branch lengths are proportional to the number of substitutions/site/year. Depicted at the main nodes are the time to most recent common ancestor estimates based on Bayesian coalescent analysis of the virus sequences and isolation dates without inclusion of the Bulgarian strain for which no reliable isolation date was available. Posterior probabilities are listed below the branches for supported nodes. Scale bar indicates nucleotide substitutions per site.

Initial S and L segment sequence comparison and phylogenetic analysis confirmed that all the LCMV and LCMV-like (including Dandenong and Kodoko) virus genome sequences were monophyletic and distinctly related to the other Old World arenaviruses (data not shown and [*26*]). Only fragments of the Kodoko virus genome sequence were available, but results of our analysis were consistent with the previous conclusion that this virus is distinct from LCMV. The S and L segment sequences of all LCMVs (including Dandenong) were distributed in 3 (L segment) or 4 (S segment) different genetic groups or lineages (Figures 1, 2). High levels of virus genetic diversity (Table 2) and protein amino acid differences were found within and between the virus lineages. Up to 18% nucleotide divergence was observed within the S segment lineages, and 22%–25% divergence between 4 characterized lineages (Table 2). Similarly, up to 25% nucleotide divergence was observed within the L segment lineages, with 27%–28% between the 3 currently identified lineages. This nucleotide divergence translates to 18%, 13%, 10%, and 6% divergence in the amino acid sequences of the Z, L, GPC, and NP proteins, respectively. While this level of diversity is considerable, it is comparable to that observed in Lassa virus (LASV), another Old World arenavirus (*27**,**28*).

**Table 2:** Genetic diversity of lymphocytic choriomeningitis virus strains*

Diversity	RNA, nt			Proteins, aa			
L	S	Z	L	GPC	N
Overall	22	19		18	13	10	6
Within lineage I	19	15		15	11	6	4
Within lineage II	25	15		34	18	7	3
Within lineage III†	NA	NA		NA	NA	NA	NA
Within lineage IV‡	NA	18		NA	NA	13	5
Between lineages I and II	27	22		27	19	11	6
Between lineages I and III	28	23		19	19	11	8
Between lineages I and IV	NA	24		NA	NA	19	9
Between lineages II and III	27	22		28	19	11	6
Between lineages II and IV	NA	24		NA	NA	18	10
Between lineages III and IV	NA	25		NA	NA	19	11

*nt, nucleotide; aa, amino acid; GPC, glycoprotein precursor; NA, not applicable. Pairwise genetic distances (uncorrected p values) were measured eliminating all positions containing alignment gaps and missing data. †Only 1 sequence in lineage III. ‡No available L sequence in lineage IV.

The S segment tree generated from analysis of 31 virus strains (29 LCMV strains and 2 Kodoko virus strains) is shown in Figure 1. The nodes separating the 4 major lineages are highly supported (posterior probability values >95). Kodoko virus is located on an ancestral branch, sister to the monophyletic clade that contains all of the LCMV strains. Most of the LCMV strains are located within lineage I, which contains all the US strains, with the exception of the virus isolate from Georgia in 1984, the sole member of lineage III (Figure 1). Lineage I includes the classic laboratory strains, WE and Armstrong, originally isolated in the 1930s. Notably, the sequences of the low passage WE strain obtained from virus stocks lyophilized in 1950 and 1960 were identical to one another, but statistically significantly different from the WE isolate currently in use and reported in GenBank (*29*). Another related strain of LCMV may have contaminated the virus stock during the passages in mice, and it is difficult to discern which virus represents the authentic WE. Although the stocks lyophilized in 1950s and 1960s were archived several decades ago, these viruses had been passaged in a laboratory in British Columbia before their return to New York.

Lineage I also includes viruses from France, Germany, and Slovakia. No obvious correlation could be seen between phylogenetic branching pattern and virus geographic origin. In addition, although this lineage contains viruses isolated during 1935–2008, no correlation was evident between phylogenetic position and date of virus isolation. These data are consistent with a long and complex evolutionary history with frequent movement of the rodent reservoir hosts during this lengthy period.

Lineage II appeared to only contain viruses from Europe. These included the LCMV M1 and M2 viruses, which had been isolated in Japan from a laboratory mouse colony established in Paris, France, from wild-caught *M. musculus musculus* that originated in Illmitz, Austria. In addition, lineage II contained the LE strain isolated from a patient in France and the Dandenong isolate obtained in Australia from a transplant recipient with a fatal LCMV infection. This patient had received organs from a donor with travel history to the Balkans before death and organ donation (*6*). Lineage IV was solely made up of viruses isolated in Spain from wild-caught wood mice (*Apodemus sylvaticus*) (*10*).

The L segment tree generated from analysis of 18 virus strains is shown in Figure 2. The overall lineage I, II, and III groupings are comparable to those observed in the S segment tree. Lineage IV is not observed because no L segment sequences were available from strains from Spain. Again, among the multiple virus representatives within lineage I, no clear correlation is apparent between phylogenetic pattern and geographic or temporal origin of the virus isolates. Most of the differences in tree topology seen between the S and L trees involve nodes, which are not strongly supported, and appear to mainly reflect the lack of resolution in the trees. In addition, the analyses do not include identical taxa sets (S and L segment data are not available for all of the viruses). Whether the observed differences reflect RNA segment reassortment or differences in evolutionary pressures cannot be discerned from the current analysis.

The Bayesian analysis enabled estimation of the rate of evolution of the 2 genome segments of the LCMV and Kodoko virus sequences. The molecular evolutionary rate for the S segment was estimated to be 3.3 × 10^–4^ substitutions/site/year with 95% highest posterior density of 1.4 × 10^–4^ to 5.2 × 10^–4^. Similarly, the molecular evolutionary rate for the L segment was 3.7 × 10^–4^ substitutions/site/y (95% highest posterior density of 1.2 × 10^–7^ to 8.6 × 10^–4^). These rates are similar to those found for other negative-stranded RNA viruses, such as the calculated rates for Rift Valley fever virus: 3.9 × 10^–4^, 3.6 × 10^–4^, and 2.8 × 10^–4^ for the S, M, and L segments, respectively (*30*).

The Bayesian analysis also addressed the question of how recently viruses may have shared a common ancestor. The most recent common ancestor for LCMV S- and L- genome RNA segments was estimated to be 1,235 and 5,142 years ago, respectively. These data indicate that LCMV is quite ancient, and the extensive diversity of the virus has accumulated over the past 1,000–5,000 years. Also, despite similar rates of evolution, the evolutionary history of the L segment appears to be more complex and can be traced back substantially longer than that for the S segment.

**Figure A1:**
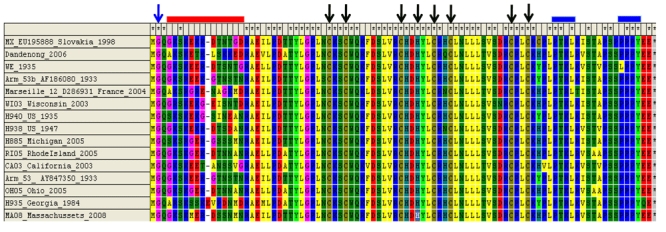
Schematic of lymphocytic choriomeningitis virus (LCMV) Z open reading frame.The N-terminal myristoylation site (*31*), RING motif, and late domains are all highly conserved among the LCMV strains analyzed. Unexpectedly, the Z protein of the H935_Georgia_1984 virus strain is 1 aa longer than all the others.

**Figure A2:**
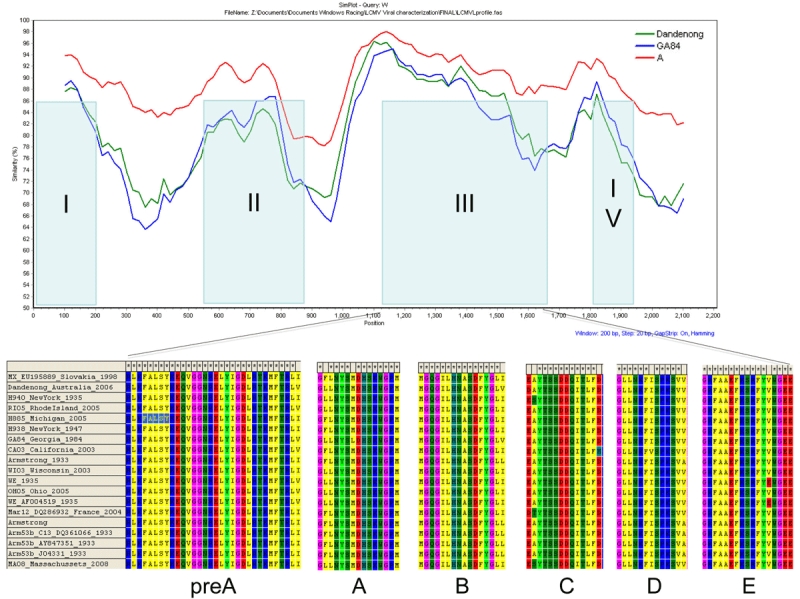
Schematic of lymphocytic choriomeningitis virus (LCMV) L open reading frame. The previously identified domains and catalytic core motifs of the LCMV L polymerase (*32*) were found to be highly conserved among all strains analyzed. Dandenong, Dandenong_Australia_2006 virus strain; GA84, H935_Georgia_1984 virus strain; A, all influenza virus type A strains.

**Figure A3:**
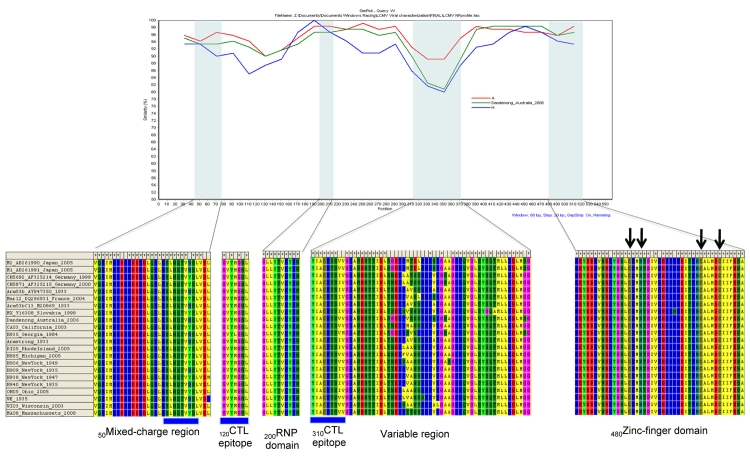
Schematic of lymphocytic choriomeningitis virus (LCMV) nucleoprotein open reading frame. Nucleoprotein protein motifs (*33*) were found to be highly conserved among all strains analyzed. A, all influenza virus type A strains.

**Figure A4:**
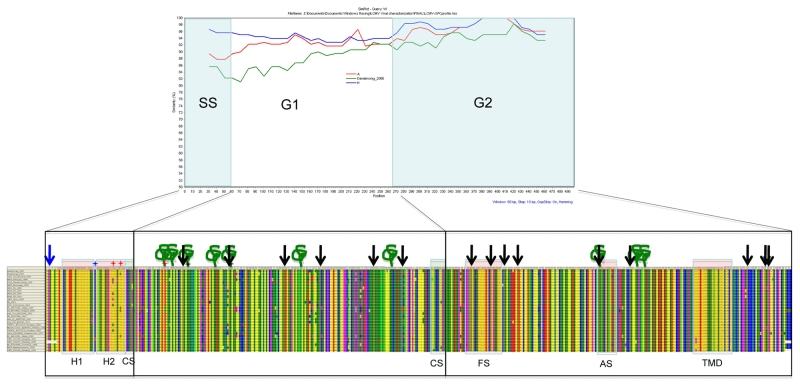
Schematic of lymphocytic choriomeningitis virus (LCMV) GPC open reading frame. The protein motifs previously identified in the Armstrong strain, such as the 2 hydrophobic domains in the signal peptide, the myristoylation site in G2, and most of the potential glycosylation sites found in other arenaviruses, are well conserved (*34*).

The protein sequences and various motifs of the diverse LCMV strains were analyzed in detail. The N-terminal myristoylation site, the RING motif, and late domains in the Z protein are all highly conserved (*31*) (Figure A1). The previously identified domains and catalytic core motifs of the LCMV L polymerase (*32*) and NP motifs (*33*) were also highly conserved among all strains analyzed (Figure A2 and Figure A3). The GPC protein motifs initially identified in the LCMV Armstrong strain, such as the 2 hydrophobic domains found in the signal peptides, the myristoylation site G_2_, and most of the predicted glycosylation sites found in other arenaviruses are well conserved (*34*) (Figure A4).

## Discussion

The primary host of LCMV is thought to be the house mouse. Three house mouse complexes within the genus *Mus* (*castaneus, domesticus*, and *musculus*) are generally recognized; however, their taxonomic rank (i.e., species vs. subspecies) has been debated extensively (*35*). Recent studies have led to the common view that house mice consist of 3 *M. musculus* subspecies, namely *M. m. musculus, M. m. domesticus, and M. m. castaneus*, that share a common ancestor ≈1 million years ago (*36*). Two of these, *M. m. domesticus* and *M. m musculus*, have been associated with LCMV infection.

*Mus* genus rodents have evolved over the past 7 million years during successive bursts of radiation and, on the basis of phylogeographic and fossil data, likely originated in Asia; the oldest *Mus* fossil recorded in Pakistan dates from the Late Miocene *37*). House mice spread to the Mediterranean Basin around 8,000 bc, and throughout the rest of Europe around 1,000 bc (*38*). The long history of association of house mice with human activity has led to their now global distribution by shipping and other commercial transport. House mice of *M. m. domesticus* descent (*35*) were first introduced to the Americas in the early 16th century aboard the ships of Spanish and Portuguese explorers and Conquistadors, and arrived in North America ≈100 years later with the French fur traders and the English colonists. Movement within the continental United States in more recent times was probably facilitated by modern means of trade and travel.

The high genetic diversity of LCMV and the lack of clear correlation of virus genetic lineages to particular geographic locations likely reflect the long and complex phylogeographic history of the house mouse host. Virus lineages I–III have all been associated with severe human disease, and lineages I and II have been directly linked to *M. musculus* rodents. Greater genetic diversity exists in Europe relative to the United States, with lineages I, II, and IV detected in Europe, but only lineages I and III in the United States. This finding may be the result of *M. musculus* mice, and presumably LCMV, having a much longer history in Europe (particularly around the Mediterranean) relative to the comparatively recent introduction of house mice into North America. In this context, it is noteworthy that LCMV viruses in lineage IV consist solely of isolates from wild-caught *Apodemus sylvaticus* mice from Spain (*10*). The close relationship between the *Mus* and *Apodemus* genera has been well-documented previously (*35*).

Given the proposed Asian origin of house mice, one would speculate that perhaps even greater LCMV genetic diversity may be found in mice of Middle Eastern or Asian origin. Unfortunately, no such LCMVs were available to test this hypothesis. Notably, the isolation of Kodoko virus from *M.* (*Nannomys*) *minutoides* (*9*) correlates with this conclusion because it forms a distinct phylogenetic lineage separate from the LCMV lineages. This species, in the subgenus *Nannomys,* is thought to be an offshoot of the Asian mice radiation that occurred around the beginning of the Pliocene ≈5 million years ago (*39*).

House mice have presumably been introduced into the United States on many occasions since the initiation of shipping traffic between the Old and New Worlds in the early 16th century. The lack of geographic correlation with LCMV genetic groupings, would suggest that traffic of LCMV-infected house mice has occurred frequently, both to the United States from the Old World and within the United States. This movement can also include the commercial traffic of LCMV-infected pet rodents (*5**,**40*). In the United States, no single genetic variant of LCMV dominates, although lineage I viruses appear to be the most frequently sampled. Similarly, there is no geographic clustering of different LCMV lineages within Europe consistent with extensive movement of the house mouse host.

Despite the generation of a large number of complete genome sequences for a diverse array of LCMV isolates, defining LCMV species solely on the basis of molecular data remains difficult. The current conservative approach is to consider all 4 identified lineages as being variants of LCMV. Although this approach would constitute a highly diverse species, it would be similar to the high genetic diversity observed within Lassa virus, another Old World arenavirus (*26**,**27*). Alternatively, it may be that the 4 LCMV lineages will be redefined as separate virus species; but that will require more detailed data regarding the virus host, serologic properties, and ecologic niche of these viruses. The diversity of LCMVs we describe has practical implications for the design of molecular diagnostic assays for screening of meningitis cases, tissue transplant materials, and the pet trade.
